# Cardiac Imaging in Patients After Fontan Palliation: Which Test and When?

**DOI:** 10.3389/fped.2022.876742

**Published:** 2022-05-16

**Authors:** Paolo Ciliberti, Paolo Ciancarella, Pasqualina Bruno, Davide Curione, Veronica Bordonaro, Veronica Lisignoli, Mario Panebianco, Marcello Chinali, Aurelio Secinaro, Lorenzo Galletti, Paolo Guccione

**Affiliations:** ^1^Department of Cardiac Surgery, Cardiology, Heart and Lung Transplantation Bambino Gesu' Children's Hospital, IRCCS, Rome, Italy; ^2^Advanced Cardiothoracic Imaging Unit, Bambino Gesù Children's Hospital, IRCCS, Rome, Italy

**Keywords:** congenital heart disease, Fontan operation, echocardiography, cardiac CT, cardiac MRI

## Abstract

The Fontan operation represents the final stage of a series of palliative surgical procedures for children born with complex congenital heart disease, where a “usual” biventricular physiology cannot be restored. The palliation results in the direct connection of the systemic venous returns to the pulmonary arterial circulation without an interposed ventricle. In this unique physiology, systemic venous hypertension and intrathoracic pressures changes due to respiratory mechanics play the main role for propelling blood through the pulmonary vasculature. Although the Fontan operation has dramatically improved survival in patients with a single ventricle congenital heart disease, significant morbidity is still a concern. Patients with Fontan physiology are in fact suffering from a multitude of complications mainly due to the increased systemic venous pressure. Consequently, these patients need close clinical and imaging monitoring, where cardiac exams play a key role. In this article, we review the main cardiac imaging modalities available, summarizing their main strengths and limitations in this peculiar setting. The main purpose is to provide a practical approach for all clinicians involved in the care of these patients, even for those less experienced in cardiac imaging.

## Background

The Fontan operation represents the final stage of a series of palliative surgical procedures for children born with complex congenital heart disease, where a “usual” biventricular physiology cannot be restored (1). Unlike the normal cardiovascular system, where pulmonary and systemic circuits are connected in series with a double propelling pump (the right and left ventricles), after this surgical palliation there is a direct connection of the systemic venous returns to the pulmonary arterial circulation without an interposed ventricle (2). In this unique physiology, the absence of a subpulmonary ventricle results in systemic venous hypertension for propelling blood through the pulmonary vasculature (2, 3). Therefore pulmonary vasculature resistance has a key role in determining adequate ventricular preload and maintaining an adequate cardiac output in these patients (2, 4). In effect modulation of PVR may provide a benefit in this peculiar setting (3, 5).

Although the Fontan operation has dramatically improved survival in patients with a single ventricle congenital heart disease (6–8), significant morbidity is still a concern (9, 10).

Chronic elevation of systemic venous pressure, required to maintain transpulmonary blood flow, leads to a multitude of complications. Furthermore, these patients experience a decreased exercise capacity due to chronic low cardiac output as a consequence of inability to adequately increase it during exercise mainly because of chronotropic incompetence (11).

In addition, as a consequence of improved survival of these patients, the incidence of the so-called “Failing Fontan” is increasing. The “Failing Fontan” is a status characterized by progressive clinical deterioration and reduced health-related quality of life (10). Multiorgan dysfunction, peripheral edema, ascites, abnormal protein turnover and liver dysfunction are in fact the main features of Fontan Failure. Its pathogenesis is still not completely understood, and once established, the outcome is poor and heart transplant is the only possible treatment. Hence, early diagnosis and cardiac transplant listing are crucial in this setting.

Due to these significant possible complications, patients who undergo the Fontan procedure need close clinical and imaging monitoring, where cardiac exams play a key role (12, 13).

The aim of this review is to highlight the information that can be obtained with each imaging modality and to illustrate their weaknesses in this peculiar setting.

## What Is The Fontan Circulation?

Nearly 10% of congenital heart defects belong to the group of functionally univentricular hearts (3). This condition is usually characterized by the presence of only one well-developed ventricle able to sustain cardiac output. Less frequently, there could be two well-developed ventricles but with a major intracardiac shunt that makes their separation not achievable. In any case, there is a “single” available ventricle that has to maintain both the systemic and the pulmonary circulations, which are not connected as usual in series but in parallel. Such a circuit has two major handicaps: arterial desaturation and a chronic ventricular volume overload (1).

The Fontan operation represents the final surgical stage in the strategy of palliation for the management of patients born with an anatomical or functional univentricular heart (1).

The idea behind this palliation is to use the only ventricle available to sustain the systemic circulation by connecting it to the aorta, while ensuring an acceptable blood flow into the lungs in a passive way, without any additional propelling source, by connecting directly the systemic veins to the pulmonary arteries. The major advantage of this strategy is the separation between the systemic and pulmonary circulation with normalization of oxygen saturation.

Since the original description (14) in the early 70s, when Fontan and Baudet performed the anastomosis of the superior caval vein to the right pulmonary artery (Glenn shunt) and the direct suture of the right atrial appendage onto the proximal right pulmonary artery in a patient with Tricuspid Atresia, several modifications have led to the contemporary total cavo-pulmonary connection (TCPC).

Currently, TCPC is achieved by a multistage surgical approach where the Fontan operation represents the last step.

In early life, the goal of the initial surgical operation (first step) is to allow for survival, providing an unobstructed systemic outlet and a controlled pulmonary blood flow. Later, total cavopulmonary connection is usually achieved using a two-stage operation. A superior cavopulmonary connection (the superior vena cava is anastomosed to the pulmonary arteries) is performed at about 6 months of age (second step). The patient remains cyanotic after this stage, because desaturated blood flow coming from the inferior caval vein is still directed to the main ventricle and then to the aorta, but ventricle loading is significantly decreased. Total cavopulmonary connection (third and last step) is performed ordinarily between 3 and 5 years of age (depending on single center preference, patient saturation, growth of vessels), connecting the inferior vena cava to the pulmonary arteries, usually by interposition of an extracardiac conduit. This leads to complete separation of the systemic and pulmonary circulations and to normalization of oxygen saturation.

In patients at high risk of system failure due to small pulmonary arteries or ventricular dysfunction, a little communication (fenestration) between the extracardiac conduit and the systemic atrium can be created, allowing a protective “right-to-left shunt”, which limits systemic venous congestion, increases ventricular preload and output, with the unavoidable drawback of some degree of arterial desaturation (15). Later, the fenestration can be closed with a device using a transcatheter approach, even if a substantial number of small fenestrations also close spontaneously during follow-up (16).

## Main Long-Term Complications In Patients With Fontan Palliation

Although when assessing long-term results of the Fontan operation several confounding factors may occur in relation to the different underlying defects and to the evolution of surgical management, long term follow-up data indicates that this operation has incredibly improved survival in patients born with “functionally univentricular hearts”. Without surgery, these patients only had a 10% chance of survival beyond the first year of life (17), whilst current survival rate after Fontan surgery seems to be of 74, 61, and 43% at 10-, 20-, and 30-years, respectively (6).

Therefore, nowadays the main issue in these patients is the significant morbidity consequent to the Fontan operation. Several complications occur late after surgery, and are almost unavoidable, mainly due to the systemic venous hypertension required to propel blood into the system (3). Potential complications are various and the most frequent are arrhythmias, thromboembolic events, cyanosis, hepatic dysfunction/cirrhosis, protein-losing enteropathy (PLE), and plastic bronchitis (10).

With the “Classic Fontan” operation, where the right atrium was incorporated in the Fontan circulation, progressive atrial dilatation was a major issue, causing atrial arrhythmias and thromboembolic events. The incidence of these adverse events has been significantly reduced by the evolution of the surgical technique. Nevertheless, arrhytmias can still occur even with TCPC, in particular supraventricular tachycardia, which is more frequent with the aging of patients and may negatively affect the hemodynamics of the Fontan circulation, also predisposing to thrombotic events. The main mechanism of formation are atrial re-entry microcircuits (18) which can be favored both by intrinsic alterations of myocardial tissue (19) and by the presence of multiple atrial sutures and surgical anastomoses.

Thromboembolic events can also occur and remain one the main causes of death in this setting (20). Predisposing factors are reduced cardiac output, abnormal flow in the systemic and pulmonary veins, atrial dilation and arrhythmias. At the same time, a reduction of coagulation factors, due to liver failure and PLE, can sometimes induce an increased hemorrhagic risk.

The increased systemic venous pressure leads to elevated pressure and congestion in the hepatic veins as well, causing pathological changes in the liver. Congestion, dilation of bile ducts, fibrosis, and cirrhosis can occur, exposing these patients even to liver malignancy (21, 22).

Additionally, the increased central venous pressure may induce recanalization of embryologically preformed and obliterated vessels, with the development of systemic to pulmonary venous collaterals that can lead to systemic desaturation and worsening of ventricular function (3).

The elevated pressure in the superior vena cava is transmitted to the thoracic duct as well, leading to impaired and abnormal lymphatic drainage, which can cause two rare but extremely dangerous complications: PLE and plastic bronchitis. Leakage into the intestine causes PLE, which represents the most frequent lymphatic problem in this setting (23). PLE implies an abnormal loss of proteins in the intestinal lumen secondary to intestinal lymphangiectasia with leakage of lymphocytes, chylomicrons and serum proteins, such as albumin and immunoglobulin, resulting in edema, ascites and immunodeficiency. Lymphatic leakage into the bronchi leads to the development of plastic bronchitis (3). This is an extremely uncommon but dreadful complication, where the formation of bronchial casts in the airway can cause bronchial obstruction and asphyxia. Casts are made up of cells and inflammatory products or of mucin and fibrin. The exact mechanism of cast formation, which is anyway driven by increased lymphatic pressure, is quite unclear.

Moreover, over time the systemic ventricle may develop systolic and/or diastolic dysfunction because of the abnormal hemodynamic condition and ventricular morphology (24). Ventricular dysfunction can be triggered both by the congenital malformation itself, even before surgical treatment, and by the abnormal working and loading ventricle conditions at the different stages of palliation. The congenital malformation itself may predispose to ventricular dysfunction especially in the presence of a morphologic right ventricle, which is not suited for and may fail after some years of systemic loading. In addition, a tricuspid valve often poorly tolerates the initial volume overload and the systemic afterload, leading to valve regurgitation and further volume overload (1). During the first months after birth, the single ventricle is always subjected to volume overload, having to sustain both the systemic and pulmonary circulations. This volume overload leads to dilation, spherical remodeling and eccentric hypertrophy. The preload to the ventricle is then acutely reduced, firstly at the time of the Glenn shunt and later at the time of the Fontan operation. The ventricle thus goes from being overloaded and overstretched to being significantly underloaded. This change in loading conditions can lead to systolic and diastolic dysfunction. Furthermore, the ventricle may enter a vicious cycle whereby diastolic impairment can further increase pulmonary vascular resistance, resulting in worsening of ventricular filling and therefore declining systolic function and cardiac output (24).

## How To Image The Fontan Circuit?

### What We Need to Know?

In a patient who underwent the Fontan operation, routine follow up must check that the palliation system is working properly. Early signals of failing should be promptly detected by imaging, both for planning a circuit optimization if possible and to lead to an early transplant listing when perfectioning can not be achieved.

The main goals of a comprehensive assessment are the ventricle and atrioventricular valve function, the patency of the Fontan pathway (IVC to PA conduit, Glenn Shunt and pulmonary arteries), and the presence of collateral flow (12). Ruling out an increase in pulmonary vascular resistance, and the presence of extracardiac issues (in particular liver dysfunction/malignancy and lymphatic disorders) are the other targets of a complete evaluation (12). Any other anatomical issue such as residual systemic obstruction (residual coarctation, ventricular septal defect stenosis, pulmonary veins compression or stenosis) must also be identified. If these lesion are amenable to correction, the palliation system efficiency may be significantly improved.

The different modalities have have their own strengths and weaknesses (Table 1) and they should be used in a complementary fashion to accurately assess anatomy, hemodynamics, and prognosis in these patients.

**Table 1:** Schematic summary of main strenghts and weaknesses of each imaging modality.

	**ECHO**	**CMR**	**CCT**	**CATH**
Easiness of use	**++++**	**++**	**+++**	**+**
Invasiveness/harmfulness	**+**	**++**	**+++**	**++++**
Anatomical assessment	**++**	**+++**	**++++**	**+++**
Functional assessment	**++**	**+++**	**++**	**++++**
Possibility of intervention	**/**	**/**	**/**	**++++**
Extracardiac assessment	**+**	**++++**	**+++**	**+**

### Strenghts and Weaknesses of the Main Imaging Modalities

#### Echocardiography

Echocardiography (ECHO) is the most used imaging modality in patients with congenital heart disease. It is an unequaled image modality in terms of availability, cost and ease of use, being possible even at the patient's bedside.Therefore, it remains the first-line imaging modality even in this peculiar setting (25).

Nevertheless, as patients grow into adulthood, image quality is often limited due to diminishing acoustic windows. Multiple factors may be implicated, such as higher BMIs and chest wall deformity, particularly in patients who underwent multiple cardiac surgical operations (26). As a consequence, in adult Fontan patients ECHO may provide incomplete information, often targeted to ventricular and valvular function.

Fontan pathway and pulmonary arteries may sometimes be visualized in case of good acoustic windows. Nevertheless, CCT or CMR should be performed to rule out any issues with certainty, allowing for unrestricted visualization of all structures (13).

Regarding ventricular function, it is well known that single ventricle function is not easy to assess, particularly when the single ventricle is a morphologic right ventricle. Agreement between echocardiographic and CMR evaluation of systolic function improves with experience in this setting but remains suboptimal. Especially when used as a test for assessing poor RV function, ECHO shows a high sensitivity, but specificity is heavily influenced by operator experience, with the risk of a relatively high rate of false positives (27). Regarding diastolic function, which is frequently impaired in these patients, it is important to know that the interpretation of standard parameters (mitral valve inflow velocities, and E/E′ratio using tissue doppler modality) in single ventricle physiology may be challenging, and standard values are generally not available (28). Atrial size as well, which is a commonly used parameter of diastolic function in normal heart physiology, is typically not applicable in Fontan patients. Therefore, although it is technically feasible to utilize standard parameters, assessing diastolic function in single ventricle is extremely challenging (29). Further studies are required for verification of specific parameters in this setting.

Atrio-Ventricular (AV) valve regurgitation is a common long-term complication in single ventricle circulation and ECHO remains the main imaging modality to routinely investigate valvar function. It can be performed using several imaging and Doppler parameters such as regurgitation jet area, vena contracta width, in addition to qualitative jet assessment (28).

In summary, ECHO remains the first line imaging and a reliable method for systolic ventricular function (Supplementary Video 1) and valve assessment. A full echocardiographic study should be part of every outpatient check-up, at intervals of no more than 12 months.

#### Cardiovascular Magnetic Resonance

Cardiovascular Magnetic Resonance (CMR) is the only method able to simultaneously obtain a comprehensive anatomical and functional assessment.

The advantages of this imaging modality are well recognized: the lack of invasiveness and ionizing radiation, the ability to visualize the entire cardio-thoracic anatomy with high spatial resolution and without acoustic window limitations, the opportunity to obtain hemodynamic and functional information thanks to the assessment of flows in any target vessel and to the accurate quantification of ventricular function (30). Excellent tissue characterization of the ventricular myocardium can also be achieved (30).

However, CMR also has some drawbacks, mainly represented by the long scanning times, which necessitate general anesthesia in uncooperative patients (such as under 7–8 years of age) and make the exam difficult to perform in clinically unwell subjects. In addition, image quality can be limited by arrhythmia and CMR is not suitable in patients suffering from claustrophobia and in the presence of unsafe metallic devices (13).

##### Anatomical Vascular Assessment

**Figure 1:**
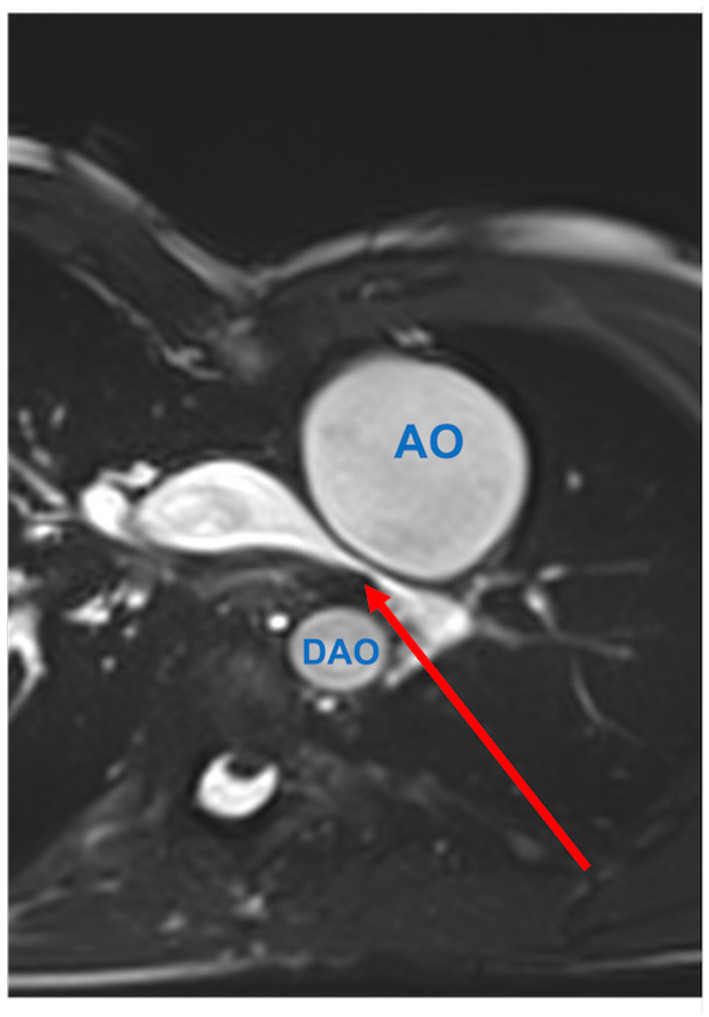
Pulmonary artery stenosis (red arrow) seen by CMR. Axial SSFP-cine view shows the left pulmonary artery is compressed between the dilated aortic root (AO) and the descending aorta (DAO).

**Figure 2:**
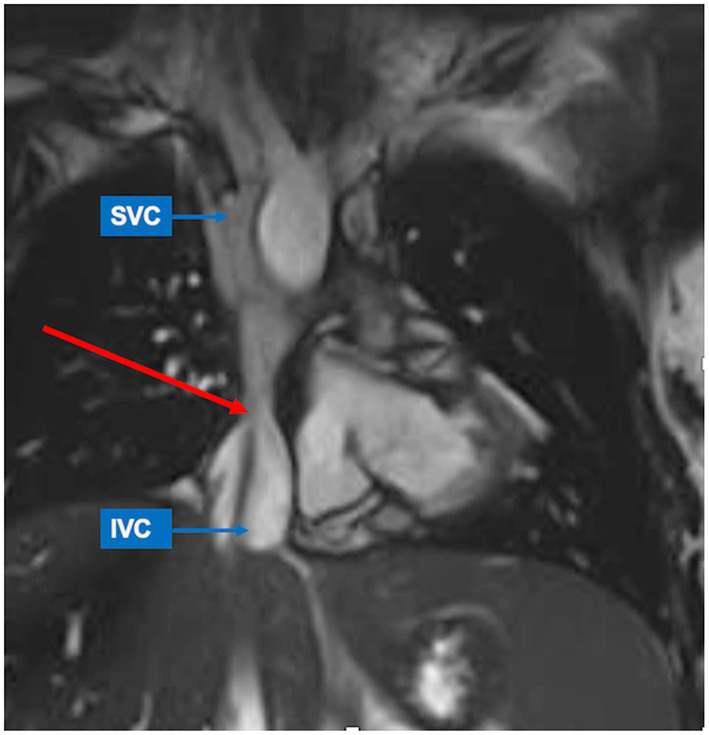
Inferior caval vein (IVC)-to-pulmonary artery stenosis (red arrow) seen by CMR. SSFP-coronal view demonstrates focal stenosis of the IVC-to-pulmonary artery pathway (red arrow). The superior caval vein (SVC) shows normal size.

The entire cardio-thoracic anatomy is well depicted with contrast-enhanced magnetic resonance angiogram (MRA) and with non-contrast ECG-gated 3D sequences. Also, cine bright-blood sequences offer a good representation of vascular anatomy thanks to high blood-tissue contrast and dynamic visualization of vessels during the cardiac cycle (Figures 1, 2). Black-blood imaging is useful when ferromagnetic devices are present, due to its lesser sensitivity to magnetic field inhomogeneity.

The Fontan pathway and branch pulmonary arteries (PAs) are carefully evaluated to rule out stenoses that could be detrimental to flow dynamics in a circulatory system without a subpulmonary pump. Central PAs can be compressed and stretched by vessel roots and neo-aorta dilatation after the Norwood operation. Residual/recurrent coarctation of the aorta after aortic arch reconstruction is another complication that occurs in these patients and can promote ventricular hypertrophy and dysfunction. Pulmonary vein compression can be harmful to passive pulmonary flow circulation and need to be excluded, especially in patients with enlarged right atrium after atriopulmonary Fontan.

##### Ventricular Evaluation and Tissue Characterization

**Figure 3:**
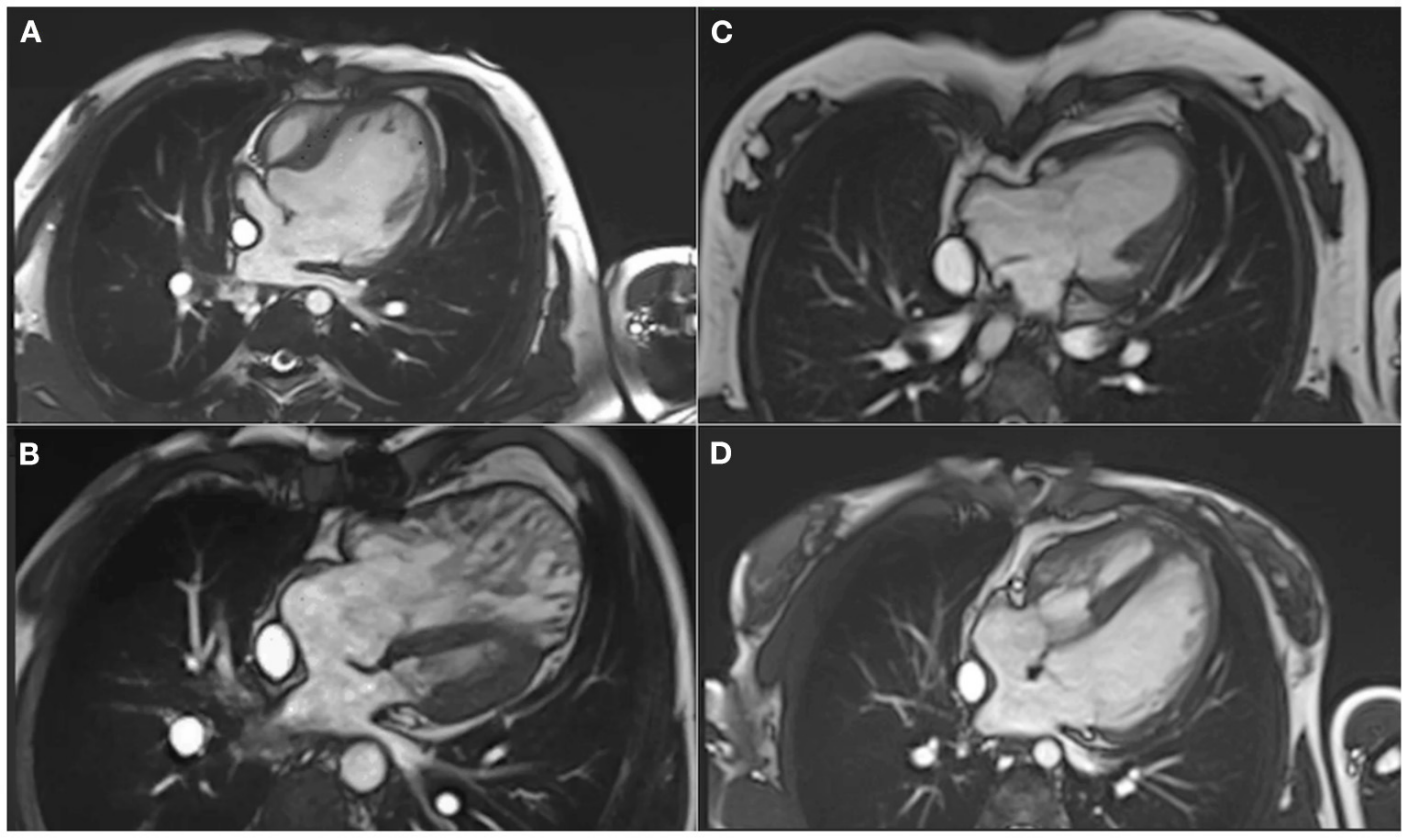
Different types of ventricle anatomy in patients with Fontan Palliation seen by CMR (4-chamber views). **(A)** Tricuspid Atresia. **(B)** Hypoplastic Left Heart Syndrome. **(C)** Double Inlet Left Ventricle. **(D)** Pulmonary Atresia and intact Ventricular Septum with hypoplastic right ventricle.

CMR is the gold standard for the assessment of ventricular size and function in univentricular hearts (31) regardless the “original anatomy” and ventricle type (Figure 3). Global and regional functional evaluation is obtained with standard cine SSFP sequences in short and long axis views. Morpho-functional parameters are calculated with dedicated post-processing software by tracing endocardial contours on a contiguous stack of short axis slices at end-diastole and end-systole, using Simpson's method. Hypoplastic ventricular chambers should be included if they contribute to systemic output, paying attention to exclude the interventricular septum from the blood pool.

Although a normal reference range for ventricular volumes in UVH has not been universally defined, serial assessment of ventricular dimensions is important because dilatation of systemic ventricle is an independent risk factor for death and transplant after Fontan palliation (32), and echocardiography systematically underestimates ventricular volumes by 30% compared to CMR (33).

Tissue characterization with late gadolinium enhancement technique allows to identify the presence and extent of myocardial fibrosis, which correlates with decreased ventricular function, increased EDVi and incidence of ventricular tachyarrhythmia (32).

##### Flow Assessment

Vessel flow analysis is routinely performed with conventional through-plane 2D phase-contrast (PC) sequences. PC can be used to assess cardiac index, calculate semilunar valve regurgitation by direct quantification or estimate atrioventricular valve regurgitation by matching antegrade systemic flow and ventricular stroke volume. Hemodynamic magnitude of a unilateral branch pulmonary artery stenosis can be evaluated by calculating disproportion of pulmonary blood flow distribution.

Flow analysis includes quantification of systemic-to-pulmonary collaterals. Elevation of central venous pressure promotes development of veno-venous collaterals which arise from superior or inferior vena cava and innominate veins, and connect to pulmonary veins or directly to the atria, thus bypassing pulmonary vascular bed with consequent cyanosis.

Aorto-pulmonary collaterals (APCs) are a response to systemic desaturation: they originate from systemic arteries (descending aorta and its branches), and supply the pulmonary circulation with high pressure flow which compete and limit deoxygenated blood flow from cavo-pulmonary connection. APCs also represent a right-to-left shunt and contribute to ventricular volume overload (34). With PC sequences, the burden of systemic-to-pulmonary collaterals can be calculated by subtracting the pulmonary arterial flow from the pulmonary venous flow or by subtracting the sum of caval flows from the aortic flow (35). Angiographic imaging can also help to identify major collateral vessels, which can represent the target for interventional procedures.

##### Extra-Cardiac Complications

**Figure 4:**
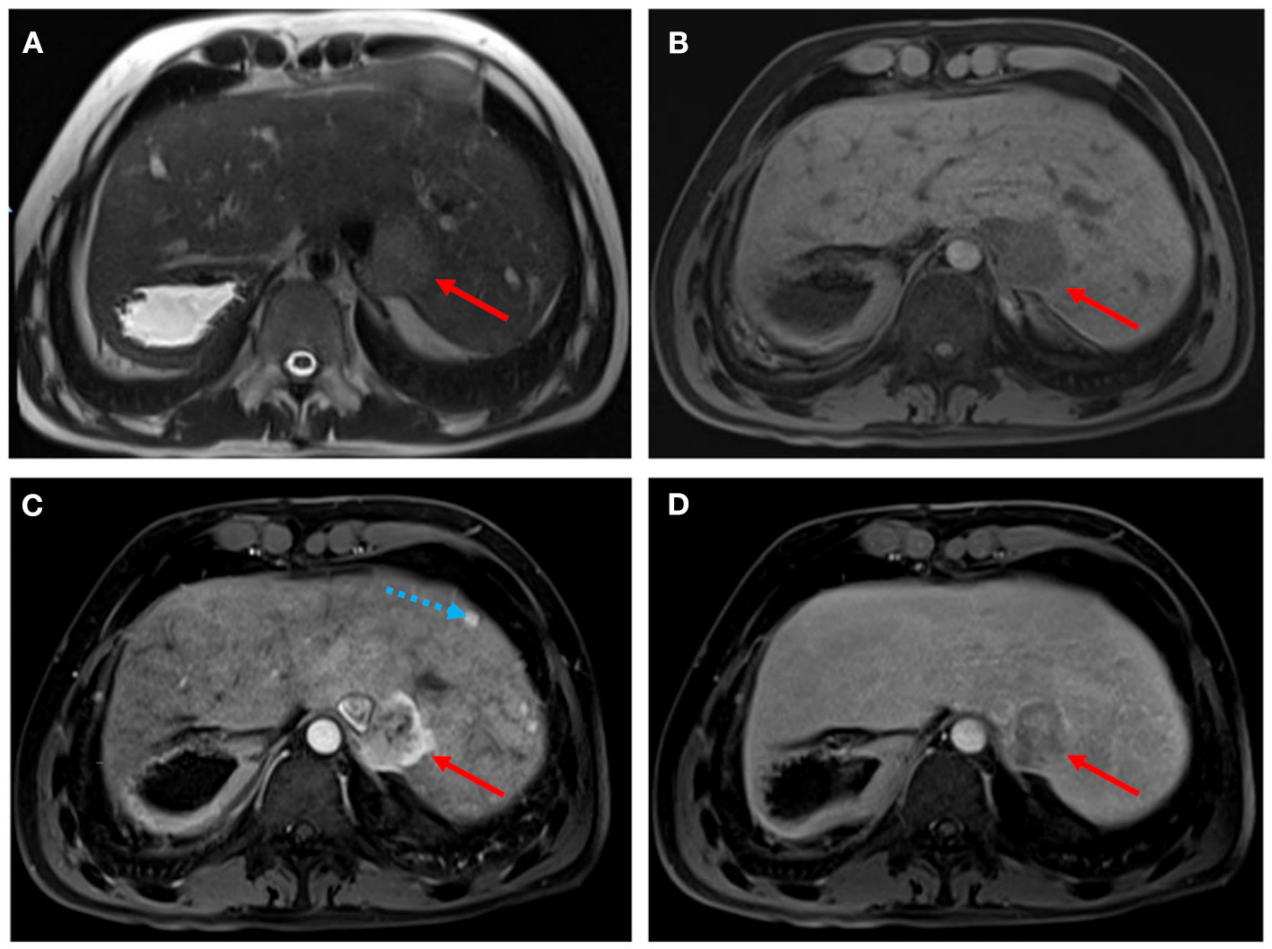
Hepatocellular carcinoma in a 30-year-old man with Fontan palliation. Axial T2- and T1-weighted MR images **(A,B)** show a 4 cm paracaval solid lesion with smooth margins and posterior hepatic capsule bulge (arrows). The tumor shows heterogeneous hyperintensity in the arterial phase of the dynamic contrast-enhanced sequence **(C)**, and typical washout in the delayed phase **(D)**. The background liver is diffusely heterogeneous after contrast injection, with multiple small arterial-enhancing foci [dotted blue arrow in **(C)**] without delayed washout, in keeping with hypervascular regenerative hepatic nodules.

Fontan-associated liver disease (FALD) represents a major non-cardiac determinant of mortality following the Fontan procedure and an emerging concern in patients with univentricular pathophysiology. Hemodynamic changes with elevated central venous pressure and diminished cardiac output promote congestive hepatopathy, resulting in progressive fibrosis/cirrhosis in response to chronic liver injury, with multiple regenerative nodules which may finally degenerate to hepatocellular carcinoma. The staging and surveillance of liver fibrosis in FALD requires a multi-modality approach, including radiological imaging. US elastography is a valuable screening investigation in identifying features of FALD and may be useful in identifying progression of FALD before biochemical hepatic dysfunction. Both magnetic resonance imaging and computed tomography provide more detailed information in delineating liver injury in FALD (Figure 4), and are more appropriate to detect and characterize focal liver lesions (36). Although there is no consensus on the surveillance plan of patients with FALD, serial follow-up of liver fibrosis and monitoring of hypervascular hepatic nodules is recommended (37).

Increased systemic venous pressure is also associated with impaired lymphatic drainage and engorgement of the lymphatic system. Patients with lymphatic perfusion abnormalities develop significant more early and late complications after Fontan completion, even in the absence of obstruction in the Fontan pathway or ventricular dysfunction. Various manifestations can occur such as protein losing enteropathy (PLE), plastic bronchitis (PB), interstitial pulmonary edema, pleuro-pericardial effusion and ascites.

**Figure 5:**
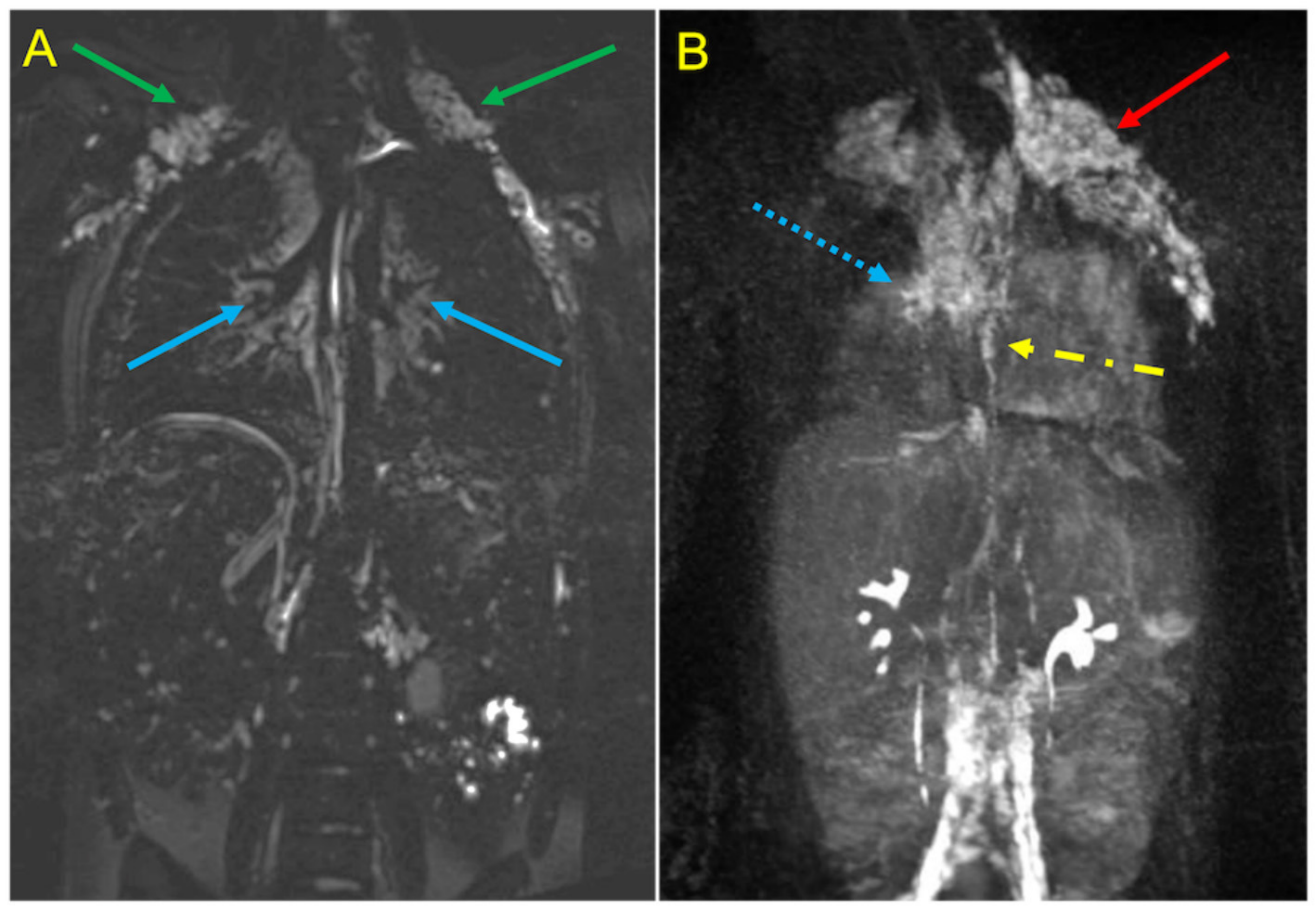
MR Lymphangiography in a Patient treated with TCPC palliation complicated by plastic bronchitis. Static T2-SPACE coronal image **(A)** demonstrates abnormal supraclavicular (green arrows) and peri-hilar (blue arrows) lymphostasis. Dynamic contrast-enhanced MIP coronal image **(B)** after intranodal contrast injection shows rapid filling of retroperitoneal lymphatics and of the thoracic duct (yellow arrow), with marked reflow into supraclavicular (red arrow) and mediastinal channels (dotted blue arrow).

PLE and PB are among the most serious complications that can occur late after Fontan Palliation. MR is able to characterize lymphatic perfusion abnormalities using static and dynamic sequences (Figure 5). Heavily T2-weighted static lymphangiography is a fast and noninvasive method to visualize the lymphatic system, define perfusion abnormalities in the thoracic and mesenteric regions and identify impaired lymphatic drainage with thoracic duct dilatation (38). Pre-Fontan assessment with T2 imaging help to identify patients with higher-grade lymphatic perfusion abnormalities who are more likely to develop post-surgical complications, improving pre-surgical risk stratification and identifying the subjects who could benefit from a lymphatic treatment (39).

Dynamic contrast-enhanced magnetic resonance lymphangiography (DCMRL) is a recently developed technique which allows a minimally invasive evaluation of the lymphatic flow pathways and their anomalies, using time-resolved MR angiography sequence after intranodal injection of gadolinium-based contrast material (40). DCMRL demonstrates abnormal pulmonary lymphatic streaming toward the peribronchial lymphatics and lung parenchyma and can be useful for planning interventional strategies of thoracic duct embolization (41).

#### Cardiac Computed Tomography

**Figure 6:**
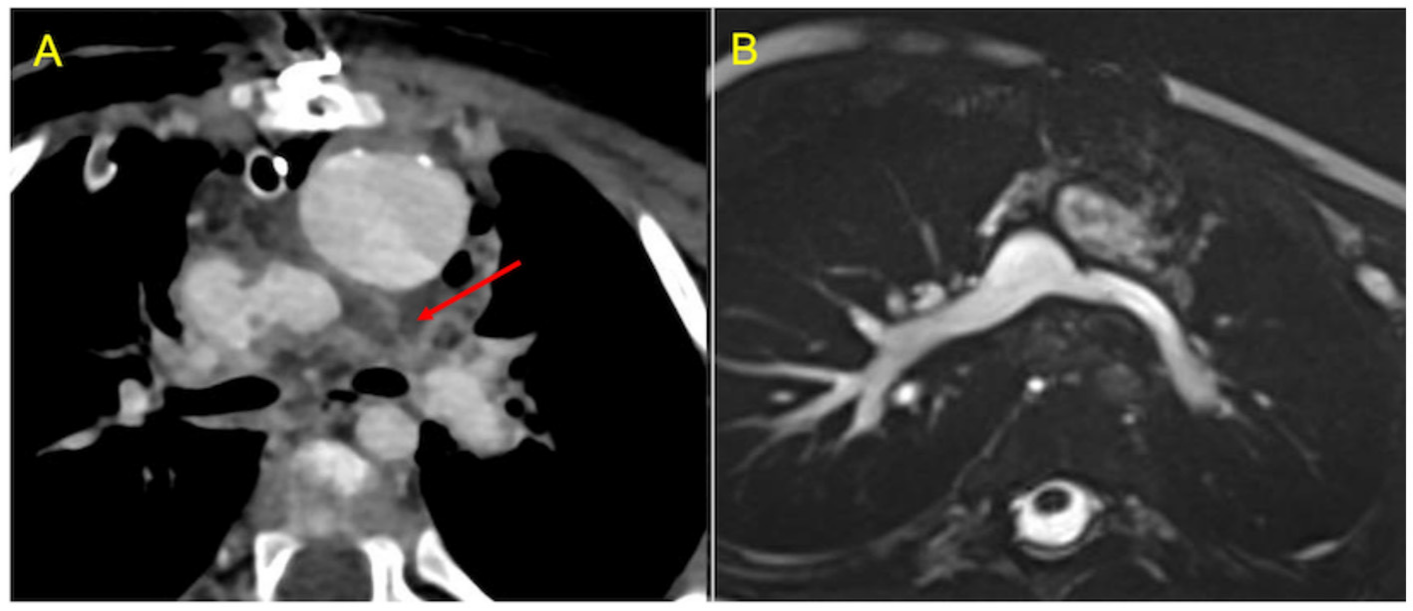
Use of CCT in the acute setting. **(A)** contrast-enhanced axial image shows early post-operative left pulmonary artery thrombosis with a filling defect extended from the cavo-pulmonary anastomosis to the left hilum (red arrow). **(B)** CMR axial SSFP view performed 2 months after surgical reintervention demonstrates vessel patency.

Cardiac Computed Tomography (CCT) is an alternative imaging modality to assess Fontan anatomy and ventricular function when CMR is contraindicated or in specific clinical conditions such as unstable and uncooperative patients, stent evaluation and acute setting (Figure 6). Thanks to its high spatial resolution and short acquisition time, CCT represents a powerful tool for a comprehensive anatomical evaluation of cardiac and extra-cardiac vascular structures, thus reducing the need for diagnostic catheterization. The main weaknesses of CCT are the lack of flow assessment, which limits functional information, relatively low temporal resolution, poor tissue contrast, and exposition to ionizing radiation (quite low with recent systems) and contrast administration (potentially causing allergic reactions and nepropathy) (42).

**Figure 7:**
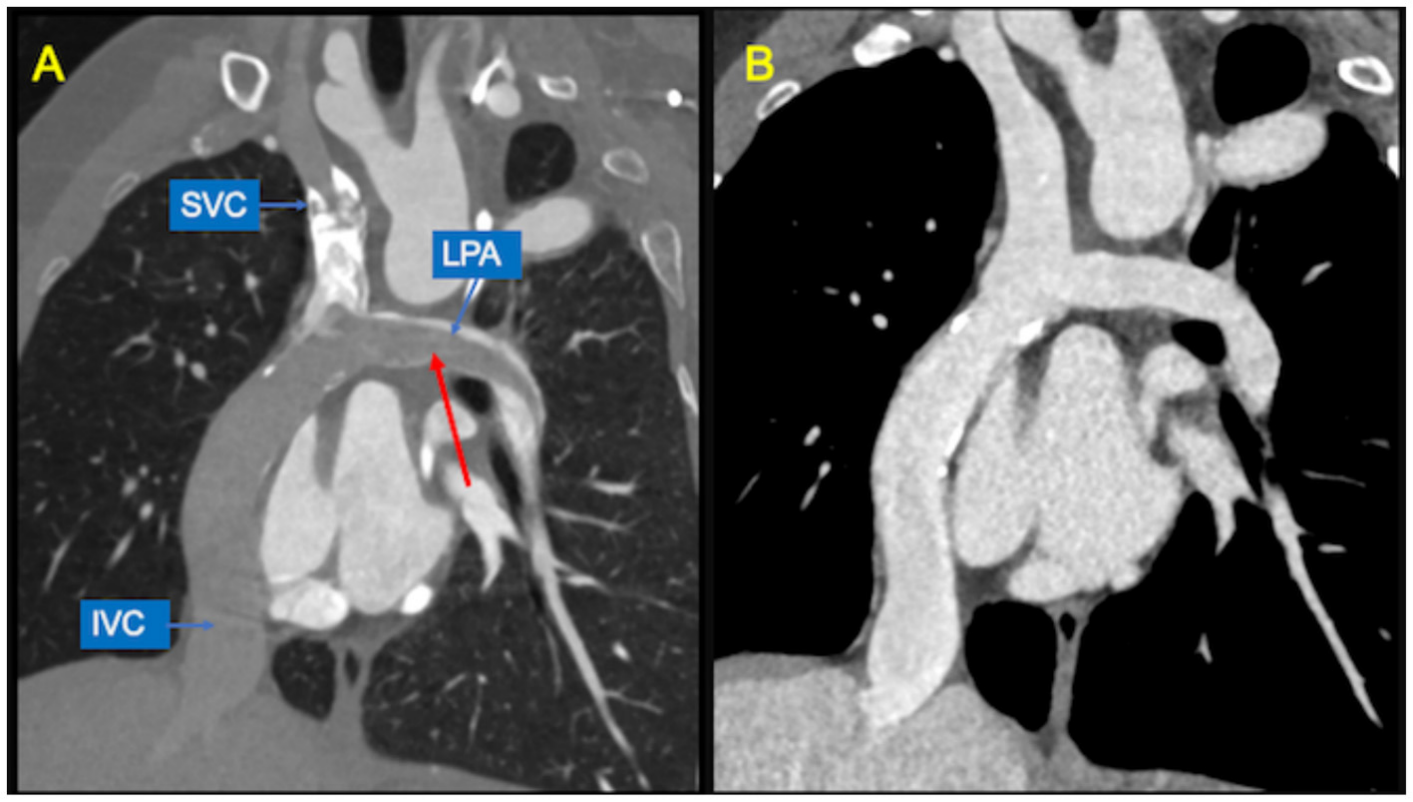
Potential pitfalls of CCT with single-injection protocol. **(A)** Coronal image shows mixing of contrast material from the superior vena cava (SVC) and unopacified blood from inferior vena cava (IVC), generating pseudo-filling defects in the left pulmonary artery (LPA) during the early arterial phase (red arrow). **(B)** The same image obtained during venous recirculation of contrast reveals regular patency of the Fontan circuit.

CCT scan in this setting can be challenging to perform. The main issue in evaluating these patients is to obtain a uniform opacification of the venous pathway and the pulmonary arteries. Typically, blood flow from the superior vena cava is directed to right lung, whereas flow from the inferior vena cava is mostly directed to the left lung. The result is a different timing of opacification of the caval veins and the pulmonary arteries. Hence, conventional contrast administration into the upper arms and first pass through the SVC and PAs generates streak artifacts and pseudo-filling defects which can mimic thrombosis (Figure 7). Various protocols have been proposed to avoid these pitfalls, such as single-injection single-phase technique with a late acquisition after 80–90 s, or a dual-injection dual-phase technique that requires contemporary use of two peripheral venous accesses (both upper and lower extremities) (43). The latter may be the preferred strategy for the assessment of pulmonary thromboembolic complications because it offers simultaneous opacification of the pulmonary arteries in the same vascular phase (44).

A systemic arterial phase acquisition is generally indicated for coronary artery imaging or when functional assessment is requested (43). Volumetric and functional data obtained with ECG-gated scan protocol shows good agreement with CMR measurement, at the expense of a substantial increase in radiation burden (45).

**Figure 8:**
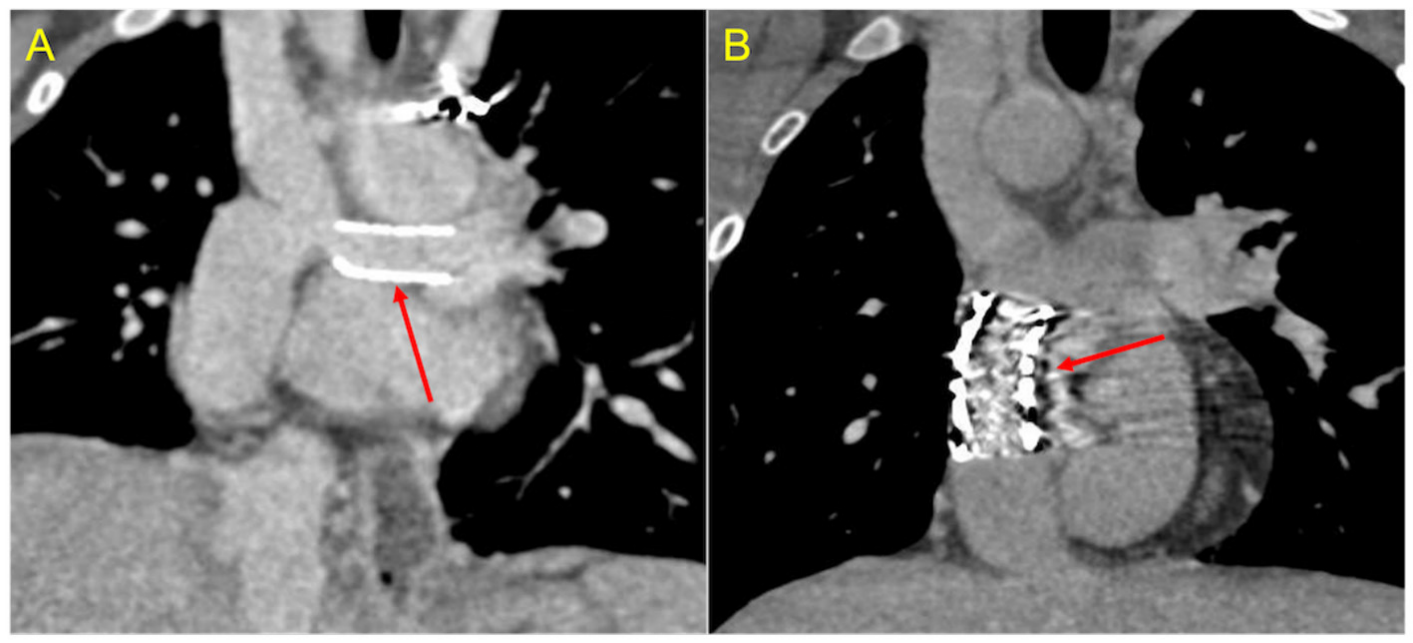
Stent assessment with CCT. **(A)** Coronal image in a Fontan patient with left pulmonary artery stenting (red arrow) illustrates how stent lumen patency can be evaluated during the venous phase with a homogeneus opacification of all vessels. **(B)** Coronal image in a Fontan patient with IVC-to-pulmonary artery conduit stenting (red arrow) demonstrates that ruling out in-stent restenosis can sometimes be difficult due to beam-hardening artifacts.

Assessment of vascular stents (Figure 8) represents another specific indication for CCT. However, beam-hardening artifacts generated by metallic material could limit the evaluation of lumen patency and in-stent restenosis caused by neo-intimal hyperplasia or stent thrombosis.

#### Cardiac Catheterization

In recent years, the use of cardiac catheterization as diagnostic imaging modality has significantly decreased, because of the advent of new non-invasive advanced imaging techniques, such as CCT and CMR.

Nevertheless, catheterization remains crucial when the likelihood of an intervention for circuit optimization is high and when specific hemodynamic parameters are needed, such as assessment of PVR, ventricular diastolic function (including constrictive and restrictive physiology) and pressure gradients (25).

Cardiac catheterization is the only method able to accurately measure end diastolic ventricular pressure, Fontan system pressure and transpulmonary gradient (46). In addition, in case of anatomical issues, such as residual aortic coartaction, or pulmonay branches or conduit stenosis, catheterization is the only technique for assessing real pressure gradients and thus the real hemodynamic significance of a lesion.

As catheter evaluation of estimated flows using the Fick principle and assumed oxygen consumption is limited and potentially incorrect (46), combined invasive catheter hemodynamics and simultaneous CMR evaluation is used in some centers to optimize vascular resistance evaluation (47, 48). This is the best method for measuring real pulmonary vascular resistance in clinical practice, especially in patients who are listed for transplantation because of a failing Fontan palliation (46). Moreover, radiation exposure remains a major drawback of pediatric and congenital cardiac catheterization. Over the recent years, many technological advances have led to faster CMR scanning time and real-time magnetic resonance imaging, allowing CMR guided cardiac catheterization (49, 50). The initial in-man experience of MRI-guided cardiac catheter interventions was halted due to MRI-compatible guidewire limitations (51). Ongoing investment and research on MRI-compatible guidewires may lead to MRI-guided diagnostic and interventional catheterization coming into the mainstream, avoiding all radiation exposure and improving soft tissue imaging (49, 51).

**Figure 9:**
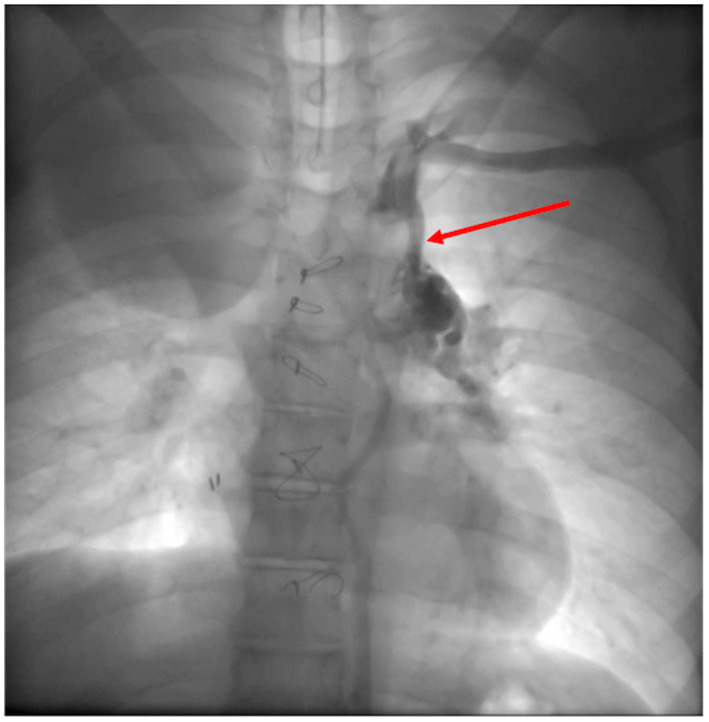
Veno-venous collateral vessel seen by Cardiac Catheterization. After injection in the left arm a large vessel (red arrow) arising from the left brachiocephalic venous system is visualized. The vessel courses inferiorly connecting to the left pulmonary veins.

Cardiac catheterization can perform treatment at almost any levels of the Fontan system, (extracardiac conduit, pulmonary arteries, superior caval vein), with angioplasty or stent placement. It is also possible to percutaneously close (if no longer needed) or create (in failing systems) a fenestration (52). If significant collateral vessels are present (Figure 9), it is possible to close them through coil injection. Treatment of these vessels increases short and medium-term survival in Fontan candidates and reduces system failure in patients after total cavo-pulmonary anastomosis.

Lymphangiography could play an important role in case of complications with a suspected lymphatic etiopathology such as protein-losing enteropathy or plastic bronchitis. Some high-volume centers are developing techniques for embolizing these periportal/peritracheal dilated lymphatic vessels with good results (53).

In summary, the main strengths of cardiac catheterization are the possibility of intracavitary pressure measurement and the possibility of interventions during the same procedure if needed. The main drawbacks include the invasive nature often requiring anesthesia or sedation, ionizing radiation exposure and contrast nephrotoxicity. Recent ESC GUCH Guidelines recommend Cardiac catheterization at a low threshold in cases of unexplained oedema, exercise deterioration, new-onset arrhythmia, cyanosis, and haemoptysis (25).

## Conclusion

Patients with Fontan physiology suffer from a multitude of unavoidable complications mainly due to the increased systemic venous pressure. Consequently, these patients need close clinical and imaging monitoring, since early recognition of failing hemodynamics can be essential to secure long-term survival.

ECHO remains the first-line method, and is mainly used for routine follow-up.

Nevertheless, nowadays cross-sectional imaging has become an essential tool in the evaluation of these patients. CMR in particular has acquired a key role in providing anatomic and functional information at the same time, overcoming the limitations of poor acoustic ECHO windows as patients become adults. CMR is also able to provide essential information about extra-cardiac complications such as liver dysfunction and abnormal lymphatic drainage. CCT may be useful when CMR is contraindicated or for anatomical assessment in uncooperative patients. Cardiac catheterization is currently reserved for measuring specific hemodynamic parameters and for those patients where an intervention during the same procedure is expected.

## Author Contributions

PCil, LG, and AS contributed to conception of the review. PCia, MC, VB, DC, PG, MP, PB, and VL wrote sections of the manuscript. PCil wrote the first draft of the manuscript. All authors contributed to manuscript revision, read, and approved the submitted version.

## Conflict of Interest

The authors declare that the research was conducted in the absence of any commercial or financial relationships that could be construed as a potential conflict of interest.

## Publisher's Note

All claims expressed in this article are solely those of the authors and do not necessarily represent those of their affiliated organizations, or those of the publisher, the editors and the reviewers. Any product that may be evaluated in this article, or claim that may be made by its manufacturer, is not guaranteed or endorsed by the publisher.

## Supplementary Material

Supplementary Video 1 Echocardiographic 4 chamber view in a patient born with Tricuspid Atresia who underwent Fontan Palliation, showing significant global systolic dysfunction.
